# Partner alcohol consumption and intimate partner violence against women in sexual unions in sub-Saharan Africa

**DOI:** 10.1371/journal.pone.0278196

**Published:** 2022-12-22

**Authors:** Richard Gyan Aboagye, Bright Opoku Ahinkorah, Charles Lwanga Tengan, Iddrisu Salifu, Henry Yaw Acheampong, Abdul-Aziz Seidu

**Affiliations:** 1 Department of Family and Community Health, Fred N. Binka School of Public Health, University of Health and Allied Sciences, Hohoe, Ghana; 2 REMS Consult Limited, Sekondi-Takoradi, Western Region, Ghana; 3 School of Public Health, Faculty of Health, University of Technology Sydney, Sydney, Australia; 4 Kumasi Center for Collaborative Research in Tropical Medicine, Kwame Nkrumah University of Science and Technology, Kumasi, Ghana; 5 Africa Centre of Excellence in Coastal Resilience, University of Cape Coast, Cape Coast, Ghana; 6 Department of Fisheries and Aquatic Sciences, School of Biological Sciences, College of Agriculture and Natural Sciences, University of Cape Coast, Cape Coast, Ghana; 7 Department of Education Studies, St. Monica’s College of Education, Mampong-Ashanti, Ghana; 8 Centre for Gender and Advocacy, Takoradi Technical University, Takoradi, Ghana; 9 College of Public Health, Medical and Veterinary Sciences, James Cook University, Townsville, Australia; University of South Florida, UNITED STATES

## Abstract

**Introduction:**

Intimate partner violence is increasingly gaining attention as the leading form of violence against women globally, particularly sub-Saharan Africa. Given that substance abuse, especially alcohol consumption has long been associated with aggressive behaviour, emotional abuse, and sexual misconduct, it is surprising that studies on the potential association between partner’s alcohol consumption and intimate partner violence are scarce. The current study seeks to fill this gap in the literature by examining the association between partner’s alcohol consumption and intimate partner violence among women in sub-Saharan Africa.

**Methods:**

Cross-sectional survey data of 89,229 women aged 15 to 49 in sexual unions from 21 sub-Saharan African countries were pooled from the Demographic and Health Surveys. Percentages with their corresponding confidence intervals (CIs) were used to present the results of the prevalence of partner’s alcohol consumption and intimate partner violence. Multivariable binary logistic regression analysis was used to examine the association between partner’s alcohol consumption and intimate partner violence. The regression analysis results were presented using adjusted odds ratio (aOR) with 95% CI. Statistical significance was set at p<0.05.

**Results:**

The pooled prevalence of partner alcohol consumption was 36.3% [36.0–36.6]. The highest prevalence of partner alcohol consumption was found in Burundi (67.1%) with Mali (3.9%) recording the lowest prevalence. Similarly, the overall prevalence of physical violence, emotional violence, and sexual violence among the women were 19.7% [19.2–20.2], 25.0% [24.5–25.5], and 9.7% [9.3–10.1], respectively. In the pooled data, women whose partners consumed alcohol were more likely to experience physical violence [aOR = 2.37, 95% CI = 2.24–2.50], emotional violence [aOR = 1.96, 95% CI = 1.86–2.07], and sexual violence [aOR = 2.03, 95% CI = 1.89–2.18] compared to those whose partners did not consume alcohol. In all the 21 countries, women whose partners consumed alcohol had higher odds for physical and emotional violence. The odds of sexual violence was higher among women whose partners consumed alcohol compared to their counterparts whose partners did not in 20 countries, except Namibia.

**Conclusions:**

We found that partner’s alcohol consumption increases women’s likelihood of experiencing physical, emotional, and sexual violence in sub-Saharan Africa. There is the need to implement behavioural change interventions targeted at male partners to reduce alcohol consumption. The findings call for the need to effectively create and organize support networks in addressing intimate partner violence among married and cohabiting women.

## Introduction

Intimate partner violence (IPV) has gained a lot of attention from human rights, health, and social experts, especially after the United Nations General Assembly passed the Declaration on the Elimination of Violence Against Women in 1993 [1]. To achieve gender equality and women’s empowerment, the Sustainable Development Goal 5 (SDG5) highlights the necessity of eliminating violence against women [2].

IPV against women is a widespread public health issue, a violation of human rights rooted in gender inequity, and a roadblock to long-term development [3, 4]. Nearly one-third (35%) of women worldwide have experienced physical and/or sexual violence by an intimate partner or sexual violence by any perpetrator in their lifetime [5]. IPV is the most common form of violence, with 30% of women in long-term relationships stating that their partner has used physical or sexual violence against them [6]. Low-and middle-income countries have a far greater incidence of IPV than high-income countries, with some of the highest prevalence estimates seen in sub-Saharan Africa (SSA) [7].

Evidence suggests that partner alcohol consumption and IPV prevalence varied significantly between countries (3–62% and 11–60%, respectively) [7]. In all 14 countries studied, partner alcohol consumption was linked to a significant increase in the likelihood of women reporting IPV. Furthermore, whereas partner alcohol use accounted for the majority of the association between alcohol use and IPV, the overall prevalence of alcohol use in a specific country also played a key role [7].

This study builds on the findings of the study by Greene et al. [7] that found partner alcohol use to be strongly associated with IPV in 14 countries in SSA by examining the association between partner’s alcohol consumption and IPV among women in SSA using data from the Demographic and Health Survey (DHS). Dissatisfaction in relationship and behavioural disinhibition are some mechanisms through which alcohol use and IPV is related according to the above study [7]. Though a similar study has been conducted in SSA, we took a holistic look at 21 countries and their respective data as well as included covariates such as partner controlling variables and decision-making variables, which were not included in the study by Greene et al. [7]. Additional feature in the present study is the segregation of the association between partner alcohol consumption and IPV per country. Findings from the study will help bring out interventions targeted at partner’s alcohol consumption as a risk factor to IPV and to help achieve the SDG target 5.2.

## Materials and methods

### Data source and study design

We utilised a cross-sectional analysis of data from the most recent DHS from 21 countries in SSA. Specifically, the data were pooled from the women’s file (individual recode). We included only countries with datasets from 2012 to 2020 and had data on domestic violence variables, partner alcohol consumption, and selected covariates. DHS used a two-stage cluster sampling method was used to recruit the respondents. A detailed sampling technique and data collection procedure have been highlighted in a previous study [8]. A total of 89,229 currently married and cohabiting women aged 15–49 years with complete cases of variables of interest were included in the final analysis (See Table 1). We relied on the Strengthening Reporting of Observational Studies in Epidemiology reporting guidelines in drafting this paper [9]. The datasets are freely accessible via this link: https://dhsprogram.com/data/available-datasets.cfm.

**Table 1:** Description of study sample.

Country	Year of survey	Weighted N	Weighted %
1. Angola	2015–16	4,783	5.4
2. Benin	2017–18	4,224	4.7
3. Burundi	2016–17	4,331	4.8
4. DR Congo	2013–14	4,689	5.2
5. Cameroon	2018	3,857	4.3
6. Ethiopia	2016	4,299	4.8
7. Gabon	2012	1,938	2.2
8. Kenya	2014	7,744	8.7
9. Liberia	2019–20	1,904	2.1
10. Mali	2018	2,941	3.3
11. Malawi	2015–16	6,419	7.2
12. Nigeria	2018	11,381	12.8
13. Namibia	2013	1,919	2.2
14. Rwanda	2014–15	3,384	3.8
15. Sierra Leone	2019	3,959	4.4
16. Chad	2014–15	4,616	5.2
17. Togo	2013–14	2,488	2.8
18. Tanzania	2015–16	3,487	3.9
19. Uganda	2016	4,715	5.3
20. Zambia	2018	3,474	3.9
21. Zimbabwe	2015	2,677	3.0
**All countries**	**2012–2020**	**89,229**	**100.0**

### Study variables

#### Outcome variables

In the present study, we considered each of the variables used to assess IPV as our outcome variables. These variables consist of physical, emotional, and sexual violence, respectively. In this study, the outcome variables were past-year experience of physical, emotional, and sexual violence. The specific questions used to measure the outcome variables were derived from the modified version of the conflict tactics scale [10, 11]. The questions used to assess the outcome variables are available in previous studies [12–14]. The responses for each question were “never” “often” “sometimes” and “yes, but not in the last 12 months”. The response options were recoded into “No [those that responded ‘never’ and yes, but not in the last 12 months’]” and “Yes [those who responded as often and sometimes’]”. The recoding of the response options have been used in previous studies [12–14].

#### Key explanatory variable

Partner’s alcohol consumption was the key explanatory variable in our study. This variable was assessed using the question “*Does (did) your (last) (husband/partner) drink alcohol*?*”*. The response options were “0 = No” and “1 = Yes”. Consistent with literature the utilised the DHS dataset [15, 16], we maintained the existing response options in the final analysis.

### Covariates

In this study, we considered seventeen variables as covariates. These variables consisted of women’s age, women’s educational level, marital status, women’s occupation, exposure to listening to radio, exposure to watching television, exposure to reading newspapers or magazines, partner’s age, partner’s educational level, justification of wife-beating, exposure to inter-parental violence, presence of partner controlling behaviour, person who usually decides on respondent’s health care, person who usually decides on respondent’s visit to family or relatives, person who usually decides on large household purchases, wealth index, and place of residence. Previous studies found these covariates as significant predictors of IPV [17–23]. These variables were also available in the DHS datasets. We maintained the existing coding in the DHS datasets for the educational level of the women and their partners, wealth index, and place of residence. However, the remaining covariates were recoded as follows; women’s occupation (not working, official, sales and services, agricultural, manual, and household and domestic/others), marital status (“married” and “cohabiting”); and partner’s age (“15–24”, “25–34”, “35–44”, and “45 and above”).

For exposure to listening to radio, exposure to watching television, and exposure to reading newspapers or magazines, the respondents whose response options to their frequency of (reading newspapers, listening to radio, and watching television) was “not all” were categorised as “Not exposed [no]” whilst those whose response options were “less than once a week”, “at least once a week” and, “almost every day” were grouped as “Exposed [yes]”.

Exposure to inter-parental violence was coded as “no” and “yes”. Each of the variables (person who usually decides on respondent’s health care, person who usually decides on respondent’s visit to family or relatives, person who usually decides on large household purchases) was coded as “respondent alone”, “respondent and partner”, “partner alone”, and “someone else/others”. Justification of wife-beatings was assessed using five questions. The respondents were asked if their husband/ partner were justified for five reasons; (i) burning food (ii) arguing with him (iii) going out without telling him (iv) neglecting the children, and (v) refusing to have sexual intercourse with him. There were three response options for each question: "no," "yes," and "don’t know." Those who responded "no" or "don’t know" had their answers recoded as "no," but those who answered "yes" had their answers maintained. After the recoding, those who answered "No" to all five questions were categorized as "not justified wife-beating [no]," while those who answered "yes" to at least one of the five questions were deemed as "justified wife-beatings [yes]".

Presence of partner controlling behaviour was measured using five out of the six questions. This is because, only Angola had data on the sixth variable (doesn’t trust her with money). The women were asked whether a current or former partner had ever engaged in the following behaviors: (i) jealousy if she talked with other men, (ii) accusations of unfaithfulness, (iii) denied her permission to meet her female friends, (iv) limited her contact with family, and (v) insisted on knowing where she was. Each question included three response options: "no," "yes," and "don’t know." Those who replied "no" or "don’t know" had their responses recoded as "No," while those who answered "yes" retained their response. Those who replied "no" to all five questions were classified as "not experienced partner controlling behaviour [no]," whereas those who answered "yes" to at least one of the five questions were classified as "experienced partner controlling behaviour [yes]". The recoded responses were used in the final analysis.

### Statistical analyses

We performed the data analyses using Stata version 16.0. The analyses were carried out in four phases. In the first phase, percentages were used to present the results of the prevalence of partner’s alcohol consumption, and physical, emotional, and sexual violence, respectively. Later, the Pearson chi-square test of independence was used to examine the distribution of the prevalence of physical, emotional, and sexual violence across partners’ alcohol consumption and studied covariates. All the statistically significant variables from the chi-square test were placed in the regression model. We employed a multivariable binary logistic regression to examine the strength of the association between partner’s alcohol consumption and physical, emotional, and sexual violence, controlling for the covariates. In the last phase of the analysis, a multivariable binary logistic regression analysis was performed and the results were segregated by country (for all the 21 countries included in the study). The results of the regression analyses were presented using adjusted odds ratios (aORs), with their respective 95% confidence intervals (CIs). We conducted a multicollinearity test using the variance inflation factor (VIF) to ascertain the existence of collinearity among the studied variables. The results showed that the minimum, maximum, and mean VIF were 1.06, 6.84, and 2.45, respectively. Hence, there was no evidence of multicollinearity among the variables studied. We applied weighting in all the analysis. The weighting was done for each individual country first before the data was appended for the countries. In doing this, the standard weighting variable for domestic violence module (d005) was divided by 1000000 to generate a new variable called “ = d005_pw”. Later, we denormalised the country level weights using the command: gen d005_pwpool = d005_pw*(total population of women; age 15–49, at the time of the survey/number of women in the resulting domestic violence subsample). After the country-level weighting generation, we appended the data for the 21 countries and used that for the final analysis.

### Ethical consideration

Ethical approval was not sought for the present study since the DHS dataset is freely available in the public domain. Further information about the DHS data usage and ethical standards are available at http://goo.gl/ny8T6X.

## Results

### Descriptive statistics

#### Prevalence of partner’s alcohol consumption and intimate partner violence among women in sub-Saharan Africa

Table 2 presents the results of the prevalence of partner alcohol consumption and IPV. The pooled prevalence of partner alcohol consumption was 36.3% [36.0–36.6]. The highest prevalence of partner alcohol consumption was found in Burundi (67.1% [65.1–69.1]) whilst the lowest prevalence was recorded in Mali (3.9% [2.8–5.3]). Similarly, the overall prevalence of physical violence, emotional violence, and sexual violence among the women were 19.7% [19.2–20.2], 25.0% [24.5–25.5], and 9.7% [9.3–10.1], respectively. Sierra Leone recorded the highest prevalence of physical (39.0% [36.3–41.7]) and emotional (39.0% [36.6–41.5]) violence, respectively. Physical violence was lowest in Togo (9.9% [8.8–11.1]) whilst emotional violence was found to be lowest in Chad (14.1% [12.4–16.1]). Sexual violence on the other hand ranged from 4.3% [3.7–5.0] in Nigeria to 20.3% [18.9–21.9] in Burundi (Table 2).

**Table 2:** Prevalence of partner’s alcohol consumption and intimate partner violence among women in sub-Saharan Africa.

Country	Partner’s alcohol consumption	Physical violence	Emotional violence	Sexual violence
Angola	41.4 [38.8–44.1]	24.1 [22.1–26.3]	24.6 [21.9–27.4]	6.6 [5.8–7.7]
Benin	27.4 [25.3–29.7]	10.9 [9.4–12.6]	29.2 [27.2–31.3]	6.1 [5.1–7.2]
Burundi	67.1 [65.1–69.1]	19.0 [17.8–20.3]	17.3 [16.0–18.7]	20.3 [18.9–21.9]
DR Congo	49.8 [47.3–52.4]	29.6 [26.9–32.4]	28.4 [25.9–30.9]	19.5 [16.4–23.1]
Cameroon	44.6 [40.8–48.4]	19.2 [16.9–21.7]	22.6 [20.2–25.2]	6.6 [5.5–7.8]
Ethiopia	29.1 [25.4–33.1]	16.8 [14.7–19.1]	20.0 [17.6–22.7]	8.5 [7.0–10.3]
Gabon	59.6 [55.9–63.3]	28.7 [25.8–31.8]	25.7 [22.7–28.9]	11.1 [9.0–13.5]
Kenya	33.4 [31.4–35.5]	22.3 [20.4–24.3]	23.7 [21.9–25.6]	9.8 [8.5–11.3]
Liberia	40.2 [36.6–44.0]	35.8 [32.2–39.6]	36.4 [32.9–40.0]	7.3 [5.2–10.3]
Mali	3.9 [2.8–5.3]	17.9 [15.9–20.0]	28.0 [25.7–30.4]	7.8 [6.5–9.3]
Malawi	29.2 [27.2–31.2]	15.3 [13.7–17.0]	22.3 [20.6–24.2]	15.5 [14.1–17.0]
Nigeria	26.3 [24.3–28.4]	11.7 [10.6–12.9]	27.5 [25.7–29.3]	4.3 [3.7–5.0]
Namibia	56.0 [52.3–59.7]	17.2 [14.6–20.3]	19.7 [16.9–22.9]	6.1 [4.5–8.3]
Rwanda	63.0 [60.0–65.9]	17.3 [15.3–19.5]	18.7 [16.7–20.9]	8.4 [6.8–10.3]
Sierra Leone	17.0 [15.2–19.0]	39.0 [36.3–41.7]	39.0 [36.6–41.5]	6.3 [5.1–7.8]
Chad	27.6 [24.3–31.1]	13.8 [11.9–15.9]	14.1 [12.4–16.1]	6.5 [5.2–8.1]
Togo	46.2 [43.4–48.9]	9.9 [8.8–11.1]	24.7 [22.6–26.9]	4.5 [3.8–5.3]
Tanzania	30.5 [28.6–32.5]	26.6 [24.8–28.4]	28.1 [25.8–30.5]	9.6 [8.7–10.7]
Uganda	41.0 [39.0–43.1]	22.9 [21.5–24.4]	30.8 [29.0–32.6]	16.9 [15.5–18.4]
Zambia	35.8 [33.6–38.1]	20.6 [19.3–22.0]	22.1 [20.6–23.7]	10.9 [9.5–12.4]
Zimbabwe	38.2 [36.2–40.1]	15.8 [14.6–17.2]	25.1 [23.6–26.9]	9.6 [8.5–10.8]
**All countries**	**36.3 [36.0–36.6]**	**19.7 [19.2–20.2]**	**25.0 [24.5–25.5]**	**9.7 [9.3–10.1]**

#### Distribution of physical, emotional, and sexual violence across partner alcohol consumption and covariates

Table 3 shows the results of the distribution of physical, emotional, and sexual violence across partner alcohol consumption and the covariates. The results showed significant relationship between partner alcohol consumption and physical, emotional, and sexual violence (p<0.05). Physical violence in particular was found to be higher among women whose partners drank alcohol (29.7%) compared to those whose partners did not drink alcohol (14.0%). Emotional violence was also more prevalent among women whose partners drank alcohol (34.2%) compared to those who had not been exposed (19.8%). Similarly, sexual violence was found to be higher among women whose partners consumed alcohol (14.6%) than among women whose partners had not been exposed to alcohol consumption (6.9%). Further, there was a significant relationship between all the covariates and physical and sexual violence at p<0.05. Also, all the covariates were significantly associated with emotional violence, except for exposure to listening to radio.

**Table 3:** Distribution of intimate partner violence across the explanatory variables.

Variable	Weighted N	Weighted %	Physical violence		Emotional violence		Sexual violence	
			Yes (%)	p-value	Yes (%)	p-value	Yes (%)	p-value
**Partner alcohol consumption**				<0.001		<0.001		<0.001
No	56,825	63.7	14.0		19.8		6.9	
Yes	32,409	36.3	29.7		34.2		14.6	
**Women’s age (years)**				<0.001		<0.001		<0.001
15–19	5,060	5.7	19.9		20.7		11.2	
20–24	14,829	16.6	23.0		25.2		11.1	
25–29	19,346	21.7	21.7		26.2		10.4	
30–34	17,435	19.5	19.8		25.8		10.0	
35–39	14,568	16.3	18.3		25.9		9.1	
40–44	10,198	11.4	17.1		23.9		7.8	
45–49	7,792	8.7	14.2		22.5		7.2	
**Women’s educational level**				<0.001		<0.001		<0.001
No education	28,463	31.9	18.5		25.1		8.5	
Primary	33,137	37.1	22.2		26.2		12.3	
Secondary	23,012	25.8	19.8		24.7		8.7	
Higher	4,617	5.2	8.9		17.1		3.3	
**Marital status**				<0.001		<0.001		<0.001
Married	70,748	79.3	18.2		24.4		9.2	
Cohabiting	18,481	20.7	25.3		27.4		11.4	
**Women’s occupation**				<0.001		<0.001		<0.001
Not working	20,520	23.0	18.8		20.4		8.7	
Official	5,410	6.1	11.6		18.9		5.5	
Sales and services	24,327	27.3	17.9		26.1		7.7	
Agricultural	31,011	34.7	22.5		27.5		12.0	
Manual	5,683	6.4	19.7		28.0		11.5	
Household and domestic/others	2,278	2.5	27.6		28.8		12.6	
**Exposure to listening to radio**				0.001		0.275		0.002
No	38,442	43.1	20.7		24.7		10.2	
Yes	50,787	56.9	19.0		25.2		9.3	
**Exposure to watching television**				<0.001		0.006		<0.001
No	55,136	61.8	20.8		25.6		11.0	
Yes	34,093	38.2	18.0		24.1		7.6	
**Exposure to reading newspaper/magazine**				<0.001		<0.001		<0.001
No	72,704	81.5	20.2		25.6		10.0	
Yes	16,525	18.5	17.3		22.6		8.4	
**Partner’s age**				<0.001		0.003		<0.001
15–24	5,265	5.9	23.0		23.1		12.4	
25–34	28,146	31.5	22.4		24.9		11.0	
35–44	29,657	33.2	19.7		26.0		9.8	
45+	26,160	29.3	16.1		24.4		7.6	
**Partner’s educational level**				<0.001		<0.001		<0.001
No education	21,943	24.6	18.2		25.0		8.4	
Primary	29,966	33.6	21.7		26.1		11.9	
Secondary	28,961	32.4	20.9		25.5		9.8	
Higher	8,359	9.4	12.4		19.6		4.9	
**Justification of wife-beatings**				<0.001		<0.001		<0.001
No	49,653	55.6	15.3		21.9		7.2	
Yes	39,576	44.4	25.2		29.0		12.8	
**Exposure to inter-parental violence**				<0.001		<0.001		<0.001
No	66,167	74.2	16.1		21.9		7.8	
Yes	23,062	25.8	30.0		34.0		15.0	
**Presence of partner controlling behaviour**				<0.001		<0.001		<0.001
No	32,329	36.2	7.3		9.2		3.4	
Yes	56,900	63.8	26.8		34.0		13.2	
**Person who usually decides on respondent’s health care**				<0.001		<0.001		<0.001
Respondent alone	17,093	19.2	24.2		30.7		12.3	
Respondent and partner	38,957	43.7	16.7		21.0		7.7	
Partner alone	32,690	36.6	20.9		26.8		10.6	
Someone else/others	489	0.5	25.1		27.1		15.4	
**Person who usually decides on visits to family**				<0.001		<0.001		<0.001
Respondent alone	17,480	19.6	23.9		30.8		12.2	
Respondent and partner	44,932	50.4	16.2		20.9		7.8	
Partner alone	26,428	29.6	22.8		28.1		11.1	
Someone else/others	389	0.4	25.2		26.3		15.2	
**Person who usually decides on large household purchases**				<0.001		<0.001		<0.001
Respondent alone	13,245	14.8	25.9		31.1		12.2	
Respondent and partner	41,188	46.2	16.5		20.9		7.7	
Partner alone	34,267	38.4	21.1		27.6		11.0	
Someone else/others	529	0.6	27.0		30.4		13.8	
**Wealth index**				<0.001		<0.001		<0.001
Poorest	16,720	18.7	22.5		27.3		10.9	
Poorer	18,037	20.2	22.7		27.1		11.2	
Middle	18,144	20.3	20.6		26.3		10.4	
Richer	17,989	20.2	18.8		24.7		9.7	
Richest	18,339	20.6	14.2		20.0		6.5	
**Place of residence**				0.024		0.004		<0.001
Urban	31,100	34.9	18.9		24.0		7.9	
Rural	58,129	65.1	20.1		25.6		10.6	

*P-values were generated from the chi-square test

#### Association between exposure to partner’s alcohol consumption and experience of intimate partner violence among women in sub-Saharan Africa

Table 4 illustrates the association between partner alcohol consumption and each of physical, emotional, and sexual violence, while controlling for the covariates. Women whose partners drank alcohol were 2 times more likely to experience physical violence compared to those whose partners did not drink [aOR = 2.37, 95% CI = 2.24–2.50]. The odds of emotional violence was higher among women whose partners drank alcohol as against those whose partners did not drink [aOR = 1.96, 95% CI = 1.86–2.07]. Also, women whose partners drank alcohol were more likely to experience sexual violence [aOR = 2.03, 95% CI = 1.89–2.18].

**Table 4:** Multivariable regression analysis of partner’s alcohol consumption and intimate partner violence among women in sub-Saharan Africa.

Variable	Physical violence aOR [95% CI]	Emotional violence aOR [95% CI]	Sexual violence aOR [95% CI]
**Partner alcohol consumption**			
No	1.00	1.00	1.00
Yes	2.37*** [2.24, 2.50]	1.96*** [1.86, 2.07]	2.03*** [1.89, 2.18]
**Women’s age (years)**			
15–19	1.00	1.00	1.00
20–24	1.28*** [1.13, 1.44]	1.29*** [1.14, 1.45]	1.05 [0.90, 1.22]
25–29	1.29*** [1.13, 1.47]	1.34*** [1.18, 1.51]	1.07 [0.91, 1.26]
30–34	1.21** [1.05, 1.39]	1.27*** [1.11, 1.45]	1.08 [0.90, 1.29]
35–39	1.14 [0.99, 1.33]	1.25** [1.09, 1.44]	1.02 [0.84, 1.23]
40–44	1.10 [0.94, 1.30]	1.11 [0.95, 1.29]	0.90 [0.73, 1.11]
45–49	0.92 [0.77, 1.10]	1.04 [0.89, 1.23]	0.89 [0.70, 1.12]
**Women’s educational level**			
No education	1.00	1.00	1.00
Primary	1.01[0.94, 1.09]	0.94 [0.88, 1.01]	1.22*** [1.11, 1.33]
Secondary	1.03 [0.94, 1.14]	1.00 [0.91, 1.09]	1.06 [0.94, 1.20]
Higher	0.73*[0.57, 0.94]	0.96 [0.79, 1.17]	0.68* [0.49, 0.94]
**Marital status**			
Married	1.00	1.00	1.00
Cohabiting	1.12*** [1.05, 1.20]	0.93* [0.87, 0.99]	0.99 [0.90, 1.08]
**Women’s occupation**			
Not working	1.00	1.00	1.00
Official	0.81* [0.69, 0.97]	1.13 [0.98, 1.30]	0.98 [0.78, 1.24]
Sales and services	0.98 [0.90, 1.07]	1.41*** [1.30, 1.52]	0.95 [0.85, 1.07]
Agricultural	1.18*** [1.09, 1.27]	1.40*** [1.31, 1.51]	1.28*** [1.15, 1.41]
Manual	1.00 [0.89, 1.13]	1.44*** [1.30, 1.61]	1.27** [1.10, 1.47]
Household and domestic/others	1.57*** [1.29, 1.92]	1.48*** [1.22, 1.80]	1.40* [1.08, 1.81]
**Partner’s educational level**			
No education	1.00	1.00	1.00
Primary	0.99 [0.92, 1.07]	0.92* [0.86, 0.99]	1.12* [1.01, 1.23]
Secondary	0.98 [0.90, 1.08]	0.91* [0.84, 0.99]	1.01 [0.90, 1.15]
Higher	0.79** [0.68, 0.92]	0.80*** [0.70, 0.91]	0.77* [0.63, 0.95]
**Partner’s age (years)**			
15–24	1.00	1.00	1.00
25–34	0.93 [0.83, 1.05]	1.00 [0.89, 1.12]	0.92 [0.79, 1.06]
35–44	0.84** [0.74, 0.96]	1.08 [0.95, 1.23]	0.83* [0.71, 0.98]
45+	0.74*** [0.64, 0.85]	1.11 [0.96, 1.27]	0.70*** [0.58, 0.84]
**Exposure to listening to radio**			
No	1.00	1.00	1
Yes	0.98 [0.93, 1.04]	1.10*** [1.04, 1.17]	1.00 [0.93, 1.08]
**Exposure to watching television**			
No	1.00	1.00	1.00
Yes	1.01 [0.93, 1.08]	1.06 [0.99, 1.14]	0.78*** [0.71, 0.85]
**Exposure to reading newspaper or magazine**			
No	1.00	1.00	1.00
Yes	0.89** [0.82, 0.97]	0.86*** [0.80, 0.93]	0.99 [0.89, 1.10]
**Justification of wife-beatings**			
No	1.00	1.00	1.00
Yes	1.48*** [1.40, 1.57]	1.15*** [1.09, 1.22]	1.48*** [1.37, 1.59]
**Exposure to inter-parental violence**			
No	1.00	1.00	1.00
Yes	1.77*** [1.67, 1.88]	1.55*** [1.47, 1.64]	1.58*** [1.47, 1.69]
**Presence of partner controlling behaviour**			
No	1.00	1.00	1.00
Yes	3.99*** [3.71, 4.28]	4.65*** [4.34, 4.97]	3.65*** [3.32, 4.00]
**Person who usually decides on respondent’s health care**			
Respondent alone	1.00	1.00	1.00
Respondent and partner	0.87** [0.80, 0.96]	0.84*** [0.77, 0.91]	0.81*** [0.73, 0.90]
Partner alone	0.86*** [0.79, 0.94]	0.84*** [0.78, 0.91]	0.89* [0.80, 0.99]
Someone else/others	0.88 [0.60, 1.29]	0.83 [0.56, 1.22]	1.10 [0.58, 2.10]
**Person who usually decides on visits to family or relatives**			
Respondent alone	1.00	1.00	1.00
Respondent and partner	0.83*** [0.76, 0.90]	0.80*** [0.74, 0.86]	0.81*** [0.74, 0.90]
Partner alone	1.06 [0.97, 1.15]	0.93 [0.86, 1.00]	0.90 [0.81, 1.01]
Someone else/others	1.05 [0.72, 1.53]	0.83 [0.57, 1.19]	1.18 [0.72, 1.93]
**Person who usually decides on large household purchases**			
Respondent alone	1.00	1.00	1.00
Respondent and partner	0.83*** [0.75, 0.91]	0.85*** [0.78, 0.93]	0.87* [0.77, 0.97]
Partner alone	0.92 [0.84, 1.01]	1.06 [0.97, 1.15]	1.05 [0.93, 1.18]
Someone else/others	1.08 [0.74, 1.58]	1.29 [0.87, 1.90]	1.03 [0.60, 1.78]
**Wealth index**			
Poorest	1.00	1.00	1.00
Poorer	1.05 [0.98, 1.14]	1.01 [0.94, 1.08]	1.08 [0.99, 1.19]
Middle	0.96 [0.88, 1.04]	0.99 [0.91, 1.06]	1.11* [1.00, 1.23]
Richer	0.86** [0.79, 0.95]	0.91* [0.83, 0.99]	1.20** [1.07, 1.36]
Richest	0.71*** [0.63, 0.80]	0.75*** [0.67, 0.84]	1.09 [0.93, 1.27]
**Place of residence**			
Urban	1.00	1.00	1.00
Rural	0.78*** [0.73, 0.85]	0.93 [0.86, 1.00]	1.03 [0.93, 1.15]
**Pseudo *R*** ^**2**^	**0.1313**	**0.1151**	**0.1018**

* *p* < 0.05,

** *p* < 0.01,

*** *p* < 0.001;

1.00 = Reference category

Table 5 presents the results on the association between partner alcohol consumption and physical, emotional, and sexual violence per country, controlling for the covariates. The results show that women whose partners drank alcohol had higher odds for experiencing physical and emotional violence across the 21 countries. Additionally, the odds of sexual violence was higher among women whose partners drank alcohol compared to their counterparts whose partners did not in 20 countries, except Namibia.

**Table 5:** Association between partner alcohol consumption and intimate partner violence among women in sub-Saharan Africa segregated by country.

Country	Physical violence	Emotional violence	Sexual violence
	aOR [95% CI]	aOR [95% CI]	aOR [95% CI]
Angola	2.51*** [2.07, 3.05]	2.02*** [1.66, 2.46]	2.38*** [1.77, 3.21]
Benin	2.28*** [1.72, 3.01]	2.79*** [2.30, 3.40]	2.49*** [1.76, 3.51]
Burundi	3.19*** [2.58, 3.93]	2.56*** [2.07, 3.15]	1.62*** [1.35, 1.94]
DR Congo	1.76*** [1.44, 2.15]	1.78*** [1.45, 2.17]	1.48** [1.17, 1.89]
Cameroon	2.91*** [2.23, 3.80]	1.96*** [1.53, 2.51]	2.46*** [1.60, 3.79]
Ethiopia	1.96*** [1.47, 2.62]	2.15*** [1.63, 2.83]	2.28*** [1.60, 3.25]
Gabon	2.09*** [1.50, 2.91]	1.94*** [1.40, 2.69]	2.43*** [1.49, 3.97]
Kenya	2.84*** [2.44, 3.59]	1.86*** [1.48, 2.34]	2.24*** [1.62, 3.10]
Liberia	2.63*** [1.90, 3.64]	1.79*** [1.29, 2.47]	2.30** [1.41, 3.76]
Mali	6.33*** [3.88, 10.33]	3.25*** [2.01, 5.24]	4.98*** [2.83, 8.78]
Malawi	3.50*** [2.73, 4.47]	2.69*** [2.19, 3.30]	2.63*** [2.10, 3.28]
Nigeria	2.73*** [2.16, 3.44]	2.51*** [2.09, 3.02]	1.75** [1.23, 2.49]
Namibia	2.38*** [1.50, 3.78]	1.55* [1.03, 2.33]	1.38 [0.70, 2.73]
Rwanda	3.42*** [2.27, 5.12]	3.09*** [2.02, 4.73]	1.79* [1.09, 2.93]
Sierra Leone	2.78*** [2.19, 3.53]	2.86*** [2.24, 3.66]	1.93** [1.32, 2.81]
Chad	3.15*** [2.14, 4.64]	2.71*** [1.87, 3.94]	1.75* [1.09, 2.81]
Togo	2.26*** [1.76, 2.89]	1.98*** [1.65, 2.38]	1.80** [1.27, 2.56]
Tanzania	2.68*** [2.27, 3.16]	2.13*** [1.81, 2.51]	2.20*** [1.76, 2.75]
Uganda	2.53*** [2.17, 2.96]	1.92*** [1.67, 2.22]	1.50*** [1.26, 1.79]
Zambia	2.72*** [2.26, 3.27]	2.62*** [2.19, 3.14]	1.93*** [1.54, 2.43]
Zimbabwe	1.87*** [1.52, 2.29]	1.62*** [1.36, 1.94]	1.35* [1.05, 1.74]

* *p* < 0.05,

** *p* < 0.01,

*** *p* < 0.001

## Discussion

In this study, we examined the association between partner’s alcohol consumption and IPV among women in SSA. We found that women whose intimate partners drank alcohol were more likely to experience all types of IPV (physical, emotional, and sexual violence). Our results corroborate the findings from previous studies [23, 26, 27], which reported that partner alcohol consumption increases women’s likelihood of experiencing violence in their intimate relationships. Our finding is consistent with that of Greene et al. [7], who argued that alcohol intake by partners is a common and well-known risk factor for IPV. Similarly, Tumwesigye et al. [24] found partner’s alcohol use as a strong predictor of physical IPV. Due to the detrimental effects of alcohol consumption, the World Health Organization [25] recommends limiting alcohol access and abusive usage due to its strong association with perpetration of physical IPV. Several factors could plausibly explain the observed association between partner’s alcohol consumption and IPV in our study. First, it is assumed that the influence of alcohol on psychological and physical capacities, as well as relationship dynamics could result in a deterioration of couple’s ability to resolve disagreements and this subsequently leads to violence perpetration in an intimate relationship. Also, intimate partner’s alcohol consumption puts financial burdens on the entire family, which subsequently creates an opportunity for partner abuse [26]. Additionally, alcohol drinking may increase misunderstanding of verbal or non-verbal cues and serves as a source of dispute in unions or relationships [27]. Furthermore, alcohol consumption by the intimate partners could have caused aggression, which might have led the partners to perpetrate violent acts against the women [28, 29].

We found that women aged 20–34 were more likely than those aged 15–19 to encounter physical, emotional, and sexual violence as a result of their partner’s alcohol usage. These findings echo those of Maguele, Taylor, and Khuzwayo [30]. In terms of marital status, our study indicated that cohabiting women were more likely to experience physical violence compared to married women. This finding accords with earlier observations from literature [31, 32], which showed that cohabiting women are more likely to experience violence. For instance, Machado et al. [32] found that dating or cohabiting partners were more likely to report physical violence than married spouses. The absence of children, emotions of higher insecurity, conflict, and interpersonal stress predominantly found among cohabiting women could have accounted for the observed finding in our study [33].

Similar to previous studies [1, 34–36], women with higher education were less likely to encounter IPV compared to those with no formal education. These differences could be explained by the fact that educated women are more likely to be and empowered compared to less educated women. In line with this, we found that higher household wealth status had an inverse relationship with physical and emotional violence against women, implying that women in well-off households have more autonomy and are less likely to experience IPV than women in poor households. In comparison to women in poor households, well-off women may be able to make better economic decisions. Another argument is that women in wealthy households have more resources at their disposal and are more empowered in their relationships, making them less vulnerable to intimate partner abuse. However, the higher odds of sexual violence among women from affluent households could be that the women were more likely to defy cultural expectations that place them below men in close relationships, especially in the African context where women are not supposed to refuse sex [37]. Therefore, any attempt by a woman to refuse sex will probably result in violence, as found in our study [37].

Justification of wife beating was also found as a predictor of IPV, an outcome which is consistent with findings from Saud et al. [38]. This justification and acceptance of IPV may lead to the reduction in the rate at which women are likely to report and seek help, and may increase the risk of women experiencing more episodes of partner abuses in the future. Additionally, the women in SSA may feel that their intimate partners can abuse them due to deep-rooted patriarchal norms and beliefs, which reinforce community tolerance for IPV [12]. Also, IPV is internalized when people have supportive views regarding it, which increases the likelihood of future perpetration as well as relational and overt victimization and this could have accounted for the observed finding in our study [12].

A statistically significant association between exposure to interparental violence and IPV was found in our study and this corroborates the findings in a previous study by Aboagye et al. [39]. This further underpins the fact that children are more likely to pick up trait or emulate the attitude of their parents or caretakers. Also, IPV as a normal component of intimate relationships by women who have experienced interparental abuse is another explanation that has been put forth, particularly in sub-Saharan African settings, where intimate relationships are constructed and dictated by cultural beliefs and concepts [39]. This finding highlights the need for early IPV detection and family support interventions and programs that can reduce children’s risk of becoming victims of IPV or abuse or perpetrators in adulthood.

Relatedly, partner controlling behavior has also been found to be a predictor of IPV. Consistent results have been reported in studies conducted in Vietnam [40] and SSA [41], where partner controlling behaviour was associated with women likelihood of experiencing IPV. Thus, the women reported IPV when their intimate partners exhibited one or more controlling behaviours. From our study, behavioral control by partners may be a significant contributor to the etiology of IPV and, thus, may need to be taken into consideration in future studies and IPV-related therapies [41].

## Strengths and limitations

The study has a number of strengths. The first major strength is the use of rigorous statistical methodologies to examine the association between partner alcohol consumption and IPV. Also, using nationally representative data assures that our findings are generalizable and repeatable throughout the 21 countries studied. Furthermore, the findings help to fill in gaps in current research on IPV by examining the relationship between partner alcohol intake and IPV. Despite the strengths, it is important to recognize that there are some inherent limitations in this study. Since the data on IPV were self-reported, there could be a possibility of recall bias, which might have influenced the findings. Given the socio-cultural norms that surround issues of IPV in some countries, the data may be prone to social desirability biases, which could affect the results of our study. It is also possible that some communities will be hesitant to report IPV in general; hence, there is the possibility of under-and over-reporting of data. In addition, the cross-sectional nature of the DHS data limits the study’s ability to draw causal inferences on the association between partner alcohol consumption and IPV.

## Conclusion

We found that partner’s alcohol consumption increases women’s physical, emotional, and sexual violence in SSA. There is the need to implement behavioural change interventions such as counseling sessions or therapy, alcohol management treatment, and batterer programs targeted at male partners to reduce alcohol consumption. Also, policies should be implemented to improve women empowerment and reduce coercive control in relationships. The findings call for the need to effectively create and organize support networks in addressing IPV among married and cohabiting women. Governments and non-governmental organizations could create it a conscious effort to make education accessible to community members, especially those living in rural and deprived communities.

## Abbreviations

aOR: Adjusted Odds Ratio
CI: Confidence Interval
DHS: Demographic and Health Survey
IPV: Intimate Partner Violence
SDG: Sustainable Development Goal
SSA: sub-Saharan Africa
VIF: Variance Inflation Factor

## References

[1] Yaya S, Ghose B. Alcohol drinking by husbands/partners is associated with higher intimate partner violence against women in Angola. Safety. 2019 Mar;5(1):5.

[2] United Nations. Transforming Our World: The 2030 Agenda for Sustainable Development; United Nations: New York, NY, USA; 2015.

[3] Chilanga E, Collin-Vezina D, Khan MN, Riley L. Prevalence and determinants of intimate partner violence against mothers of children under-five years in Central Malawi. BMC Public Health. 2020 Dec;20(1):1–4. 33267864 10.1186/s12889-020-09910-z PMC7709392

[4] World Health Organization. Violence against women: Intimate partner and sexual violence against women: Intimate partner and sexual violence have serious short-and long-term physical, mental and sexual and reproductive health problems for survivors: Fact sheet. World Health Organization; 2014.

[5] World Health Organization. Global and regional estimates of violence against women: prevalence and health effects of intimate partner violence and non-partner sexual violence. World Health Organization; 2013.

[6] Mohammed BH, Johnston JM, Harwell JI, Yi H, Tsang KW, Haidar JA. Intimate partner violence and utilization of maternal health care services in Addis Ababa, Ethiopia. BMC Health Services Research. 2017 Dec;17(1):1–0. 28270137 10.1186/s12913-017-2121-7 PMC5341201

[7] Greene MC, Kane JC, Tol WA. Alcohol use and intimate partner violence among women and their partners in sub-Saharan Africa. Global Mental Health. 2017;4. doi: 10.1017/gmh.2017.9 29230309 PMC5719482

[8] Aliaga A, Ruilin R. Cluster optimal sample size for demographic and health surveys. In7th International Conference on Teaching Statistics–ICOTS 2006 Jul (Vol. 7, pp. 2–7).

[9] Von Elm E, Altman DG, Egger M, Pocock SJ, Gøtzsche PC, Vandenbroucke JP, Strobe Initiative. The Strengthening the Reporting of Observational Studies in Epidemiology (STROBE) Statement: Guidelines for reporting observational studies. International Journal of Surgery. 2014 Dec 1;12(12):1495–9. doi: 10.1016/j.ijsu.2014.07.013 25046131

[10] Kishor S. Domestic violence measurement in the demographic and health surveys: The history and the challenges. Division for the Advancement of Women. 2005 Apr:1–0.

[11] Straus MA. Measuring intrafamily conflict and violence: The conflict tactics (CT) scales. Journal of Marriage and the Family. 1979 Feb 1:75–88.

[12] Aboagye RG, Okyere J, Seidu AA, Hagan JE, Ahinkorah BO. Experience of Intimate Partner Violence among Women in Sexual Unions: Is Supportive Attitude of Women towards Intimate Partner Violence a Correlate?. Healthcare. 2021 May: 9(5): 563–73 doi: 10.3390/healthcare9050563 34064797 PMC8151125

[13] Ahinkorah BO. Polygyny and intimate partner violence in sub-Saharan Africa: Evidence from 16 cross-sectional demographic and health surveys. SSM-Population Health. 2021 Mar 1; 13:100729. doi: 10.1016/j.ssmph.2021.100729 33511263 PMC7815814

[14] Ahinkorah BO, Onayemi OM, Seidu AA, Awopegba OE, Ajayi AI. Association between girl-child marriage and intimate partner violence in Sub-Saharan Africa: insights from a multicountry analysis of demographic and health surveys. Journal of Interpersonal Violence. 2021 Apr 9:08862605211005139. doi: 10.1177/08862605211005139 33832374

[15] Solanke BL. Does exposure to interparental violence increase women’s risk of intimate partner violence? Evidence from Nigeria demographic and health survey. BMC International Health and Human Rights. 2018 Dec;18(1):1–3. doi: 10.1186/s12914-018-0143-9 29325549 PMC5765632

[16] Islam MJ, Rahman M, Broidy L, Haque SE, Saw YM, Duc NH, Haque MN, Rahman MM, Islam MR, Mostofa MG. Assessing the link between witnessing inter-parental violence and the perpetration of intimate partner violence in Bangladesh. BMC Public Health. 2017 Dec;17(1):1–0. 28187721 10.1186/s12889-017-4067-4 PMC5303211

[17] Izugbara CO, Obiyan MO, Degfie TT, Bhatti A. Correlates of intimate partner violence among urban women in sub-Saharan Africa. PloS ONE. 2020 Mar 25;15(3): e0230508. doi: 10.1371/journal.pone.0230508 32210457 PMC7094863

[18] Abeya SG, Afework MF, Yalew AW. Intimate partner violence against women in western Ethiopia: prevalence, patterns, and associated factors. BMC Public Health. 2011 Dec;11(1):1–8. doi: 10.1186/1471-2458-11-913 22151213 PMC3252295

[19] Abramsky T, Watts CH, Garcia-Moreno C, Devries K, Kiss L, Ellsberg M, Jansen HA, Heise L. What factors are associated with recent intimate partner violence? Findings from the WHO multi-country study on women’s health and domestic violence. BMC Public Health. 2011 Dec;11(1):1–7. 21324186 10.1186/1471-2458-11-109 PMC3049145

[20] Tiruye TY, Harris ML, Chojenta C, Holliday E, Loxton D. Determinants of intimate partner violence against women in Ethiopia: A multi-level analysis. PLoS ONE. 2020 Apr 24;15(4): e0232217. doi: 10.1371/journal.pone.0232217 32330193 PMC7182270

[21] Tenkorang EY. Women’s autonomy and intimate partner violence in Ghana. International Perspectives on Sexual and Reproductive Health. 2018 Jun 1;44(2):51–61. doi: 10.1363/44e6118 30321136

[22] Yaya S, Hudani A, Buh A, Bishwajit G. Prevalence and predictors of intimate partner violence among married women in Egypt. Journal of Interpersonal Violence. 2019 Nov 13:0886260519888196. doi: 10.1177/0886260519888196 31718407

[23] Wang T, Liu Y, Li Z, Liu K, Xu Y, Shi W, Chen L. Prevalence of intimate partner violence (IPV) during pregnancy in China: A systematic review and meta-analysis. PloS ONE. 2017 Oct 2;12(10): e0175108. doi: 10.1371/journal.pone.0175108 28968397 PMC5624577

[24] Tumwesigye NM, Kyomuhendo GB, Greenfield TK, Wanyenze RK. Problem drinking and physical intimate partner violence against women: evidence from a national survey in Uganda. BMC Public Health. 2012 Dec;12(1):1–1. doi: 10.1186/1471-2458-12-399 22672439 PMC3406983

[25] WHO/LSHTM: Preventing Intimate Partner and Sexual Violence against women: taking action and generating evidence. Geneva: World Health Organization; 2010:10–78.

[26] Fawole AO, Hunyinbo KI, Fawole OI. Prevalence of violence against pregnant women in Abeokuta, Nigeria. Australian and New Zealand Journal of Obstetrics and Gynaecology. 2008 Aug;48(4):405–14. doi: 10.1111/j.1479-828X.2008.00868.x 18837847

[27] Stöckl H, March L, Pallitto C, Garcia-Moreno C. Intimate partner violence among adolescents and young women: prevalence and associated factors in nine countries: a cross-sectional study. BMC Public Health. 2014 Dec;14(1):1–4. doi: 10.1186/1471-2458-14-751 25059423 PMC4133076

[28] Gebrezgi BH, Badi MB, Cherkose EA, Weldehaweria NB. Factors associated with intimate partner physical violence among women attending antenatal care in Shire Endaselassie town, Tigray, northern Ethiopia: A cross-sectional study, July 2015. Reproductive Health. 2017 Dec;14(1):1–0. 28646921 10.1186/s12978-017-0337-y PMC5483282

[29] Laelago T, Belachew T, Tamrat M. Prevalence and associated factors of intimate partner violence during pregnancy among recently delivered women in public health facilities of Hossana town, Hadiya zone, southern Ethiopia. Open Access Libriary Journal. 2014 Oct 1;1(07):1.

[30] Maguele MS, Tlou B, Taylor M, Khuzwayo N. Risk factors associated with high prevalence of intimate partner violence amongst school-going young women (aged 15–24years) in Maputo, Mozambique. PLoS ONE. 2020 Dec 9;15(12): e0243304. doi: 10.1371/journal.pone.0243304 33296426 PMC7725391

[31] Manning WD, Longmore MA, Giordano PC. Cohabitation and intimate partner violence during emerging adulthood: High constraints and low commitment. Journal of Family Issues. 2018 Mar;39(4):1030–55. doi: 10.1177/0192513X16686132 29531425 PMC5844497

[32] Machado C, Martins C, Caridade S. Violence in intimate relationships: A comparison between married and dating couples. Journal of Criminology. 2014 Aug 28;2014.

[33] Brown SL, Bulanda JR. Relationship violence in young adulthood: A comparison of daters, cohabitors, and marrieds. Social Science Research. 2008 Mar 1;37(1):73–87.

[34] Kishor S, Johnson K. Profiling domestic violence: A multi-country study. MEASURE DHS+, ORC Macro; 2004.

[35] Wilson IM, Graham K, Taft A. Alcohol interventions, alcohol policy and intimate partner violence: A systematic review. BMC Public Health. 2014 Dec;14(1):1–1. doi: 10.1186/1471-2458-14-881 25160510 PMC4159554

[36] Zablotska IB, Gray RH, Koenig MA, Serwadda D, Nalugoda F, Kigozi G, Sewankambo N, Lutalo T, Mangen FW, Wawer M. Alcohol use, intimate partner violence, sexual coercion and HIV among women aged 15–24 in Rakai, Uganda. AIDS and Behavior. 2009 Apr;13(2):225–33. doi: 10.1007/s10461-007-9333-5 18064556

[37] Aboagye RG, Dadzie LK, Arthur-Holmes F, Okyere J, Agbaglo E, Ahinkorah BO, Seidu AA. Intimate partner violence against married and cohabiting women in sub-Saharan Africa: does sexual autonomy matter?. Reproductive Health. 2022;19(1):1–1. 35346246 10.1186/s12978-022-01382-1 PMC8962047

[38] Saud M, Ashfaq A, Mas’ udah S. Women’s Attitudes towards Wife Beating and its Connection with lntimate Partner Violence (lPV): An Empirical Analysis of a National Demographic and Health Survey Conducted in Pakistan. Journal of lnternational Women’s Studies. 2021;22(5):149–60.

[39] Aboagye RG, Seidu AA, Asare BY, Peprah P, Addo IY, Ahinkorah BO. Exposure to interparental violence and justification of intimate partner violence among women in sexual unions in sub-Saharan Africa. Archives of Public Health. 2021 Dec;79(1):1–1. 34503582 10.1186/s13690-021-00684-3 PMC8428140

[40] Krantz G, Vung ND. 2The role of controlling behaviour in intimate partner violence and its health effects: a population based study from rural Vietnam. BMC public health. 2009 Dec;9(1):1–0. 19442288 10.1186/1471-2458-9-143 PMC2689202

[41] McClintock HF, Trego ML, Wang EM. Controlling behavior and lifetime physical, sexual, and emotional violence in sub-Saharan Africa. Journal of interpersonal violence. 2021 Aug;36(15–16):7776–801. doi: 10.1177/0886260519835878 30913962

